# Pain characterization in osteosarcopenia: an exploratory study

**DOI:** 10.3389/fresc.2025.1731791

**Published:** 2026-01-26

**Authors:** Antimo Moretti, Marco Paoletta, Francesco P. Fabrazzo, Sara Liguori, Mariangela Airoma, Michele Tardugno, Francesca Gimigliano, Giovanni Iolascon

**Affiliations:** 1 Dipartimento Multidisciplinare di Specialità Medico-Chirurgiche e Odontoiatriche, Università Della Campania Luigi Vanvitelli, Naples, Italy; 2 Department of Mental and Physical Health and Preventive Medicine, University of Campania Luigi Vanvitelli, Naples, Italy

**Keywords:** osteoporosis, osteosarcopenia, quality of life, rehabilitation, sarcopenia

## Abstract

**Objective:**

To characterize pain in terms of frequency, intensity, and correlation with nutritional status, muscle mass, and physical performance in osteosarcopenic patients.

**Method:**

We included patients affected by osteosarcopenia (OSP), according to WHO criteria and EWGSOP2 guidelines. Assessments included Bone Mineral Density, Trabecular Bone Score and Appendicular Lean Mass, Handgrip Strength, Short Physical Performance Battery, and SARC-F. Pain was investigated by the Brief Pain Inventory, quality of life by the EuroQol-5D-3L, the nutritional status by the Mini Nutritional Assessment-Short Form, and the level of physical activity by the International Physical Activity Questionnaire.

**Results:**

We included 42 OSP patients with a mean age of 69.3 ± 11.3 years. Pain frequency was 78.6%. Pain severity was inversely correlated with lean mass, nutritional status, and physical performance. Pain interference correlated with impaired motor skills, balance and gait speed, and poor quality of life.

**Conclusion:**

Pain is highly frequent and moderately correlated in individuals with OSP highlighting the need for integrated interventions focused on muscle health to improve quality of life and reduce fall and fracture risks.

## 1 Introduction

Osteosarcopenia (OSP) has recently been defined as a multifactorial syndrome characterized by the coexistence of two conditions: osteoporosis (or osteopenia) and sarcopenia (1–3). While osteoporosis is a systemic skeletal disease marked by a reduction in bone mass and deterioration of bone microarchitecture (4), sarcopenia is characterized by a generalized and progressive loss of skeletal muscle mass and muscle function, resulting in an increased risk of adverse events such as falls, disability, and poor quality of life (QoL) (5, 6).

The prevalence of OSP is estimated to range between 5% and 37% among older adults living in the community (7). However, considering the global ageing of the population, the prevalence of both osteoporosis and sarcopenia is expected to rise, along with the associated risk of fragility fractures, leading to increased morbidity, disability, and mortality (8). Among the impairments associated with OSP, musculoskeletal pain is frequently reported as one of the most disabling symptoms in clinical practice. Nevertheless, pain is not typically considered a hallmark of either osteoporosis or sarcopenia, both of which are often described as clinically silent conditions. When pain is present in patients affected by these conditions, it remains poorly characterized. The underlying mechanisms contributing to pain in OSP are not fully understood and may be multifactorial, possibly involving altered biomechanics, microstructural damage, chronic low-grade inflammation, or coexisting musculoskeletal disorders (9). Furthermore, the clinical significance of pain in individuals with OSP has not been clearly established. Its potential impact on mobility, physical function, nutritional status, psychological well-being, and adherence to both pharmacological and non-pharmacological therapies is still underexplored (10). Despite the growing body of evidence highlighting the social, economic, and clinical burden of chronic pain in older adults, pain in the context of OSP is often underestimated and inadequately managed, contributing to delayed interventions and suboptimal outcomes (9). In light of these observations, our objective was to examine the frequency and intensity of pain, as well as its correlation with nutritional status, physical performance, and muscle mass within a cohort of osteosarcopenic patients.

## 2 Materials and methods

### 2.1 Patient population

We conducted an observational study including individuals selected from a database of 523 outpatients evaluated at the Physical Medicine and Rehabilitation Unit of the University of Campania “Luigi Vanvitelli” between January 2023 and October 2024. Inclusion criteria were: (a) age ≥50 years, (b) a BMD T-score <−1 standard deviation (SD) on dual-energy x-ray absorptiometry (DXA), and (c) a handgrip strength (HGS) <27 kg for men and <16 kg for women, along with an Appendicular Lean Mass (ALM) <20 kg for men and <15 kg for women. Exclusion criteria were: (a) end-stage organ disease; (b) moderate-to-severe cognitive impairment or aphasia that could interfere with adherence to the study protocol; and (c) non-cooperative behavior during clinical examination.

All participants provided written informed consent after receiving a comprehensive explanation of the study aims and procedures.

Osteoporosis and osteopenia were diagnosed in accordance with World Health Organization (WHO) criteria: osteoporosis was defined as a *T*-score ≤−2.5 SD, and osteopenia as a *T*-score between −1.0 and −2.5 SD (11). Sarcopenia was diagnosed following the criteria of the European Working Group on Sarcopenia in Older People (EWGSOP2). According to the EWGSOP2, low muscle strength is the primary characteristic of sarcopenia (HGS <27 kg for men and <16 kg for women), with confirmation by low muscle quantity (ALM <20 kg in men; <15 kg in women). Poor physical performance (SPPB ≤8) was used to identify severe sarcopenia (12, 13).

The study was conducted in accordance with the latest revision of the Declaration of Helsinki and was approved by the Ethics Committee of the University of Campania “Luigi Vanvitelli” (protocol number: 412, 30/05/2018).

### 2.2 Outcome measures

We collected sociodemographic and clinical data, including sex, age, height, weight, and body mass index (BMI). Musculoskeletal assessments included bone and muscle evaluations: BMD and ALM were measured using DXA; the Trabecular Bone Score (TBS), a DXA-derived index of bone microarchitecture, was used to complement BMD data without additional radiation exposure (14, 15); HGS was measured using a handheld dynamometer (JAMAR® Dynamometer) following standardized protocols; physical performance was assessed using the Short Physical Performance Battery (SPPB), which evaluates gait speed, chair rise ability, and balance. The SPPB is widely used to detect frailty and predict the risk of falls, disability, and mortality in older adults (2, 5). Sarcopenia risk was screened using the SARC-F questionnaire, which evaluates self-reported difficulties in strength, walking, rising from a chair, stair climbing, and fall history. Scores ≥4 indicate a higher risk of sarcopenia and warrant further evaluation (16, 17).

Musculoskeletal pain was assessed using the Brief Pain Inventory (BPI). The BPI evaluates pain severity (Severity score, BPI-SS) and interference (Interference score, BPI-IS) with daily activities and emotional well-being (18, 19). Each item is rated on a numeric scale from 0 to 10, where 0 indicates “no pain” or “does not interfere” and 10 indicates “worst pain imaginable” or “completely interferes.” The BPI-SS is calculated as the mean of four severity items, and the BPI-IS as the mean of seven interference items. Therefore, the maximum possible score for both dimensions is 10 (20).

Health-related quality of life was measured by the European Quality of Life 5 Dimensions 3 Level Version (EQ-5D-3L) instrument, which assesses five domains: mobility, self-care, usual activities, pain/discomfort, and anxiety/depression (21, 22).

Nutritional status was investigated using the Mini Nutritional Assessment-Short Form (MNA-SF), a validated screening tool for malnutrition in older adults (23, 24).

Physical activity level was measured with the short form of the International Physical Activity Questionnaire (IPAQ-SF) (25, 26). This instrument captures time spent in vigorous and moderate activity, walking, and sedentary behavior over the past seven days, with results expressed as Metabolic Equivalent of Task (MET)-minutes per week (27).

### 2.3 Statistical analysis

Statistical analyses were performed using the Statistical Package for the Social Sciences (SPSS), version 25 (IBM Corp., Armonk, NY, USA). Continuous variables were expressed as means ± standard deviations (SD) or as medians with interquartile ranges (IQR), as appropriate. Categorical variables were presented as absolute counts and percentages.

Normality was assessed using the Shapiro–Wilk test. For group comparisons, we used either the Student's *t*-test or the Mann–Whitney *U* test, after evaluating variance with Levene's test. Correlation analyses were performed using Pearson's correlation coefficient or Spearman's rank correlation coefficient, depending on data distribution. Statistical significance was set at *p* < 0.05.

## 3 Results

A total of 42 subjects were included in the study (37 females and 5 males), all diagnosed with OSP.

Socio-demographic and clinical characteristics of included patients are reported in (Table 1). These patients had a mean age of 69.3 ± 11.3 years and a mean BMI of 24.6 ± 3.5 kg/m^2^. BMI analysis revealed that only one patient was underweight, while 16 were overweight and two were classified as obese. Despite this, the mean MNA-SF score was 11.4 ± 2.3, indicating a mild risk of malnutrition.

**Table 1:** Socio-demographic and clinical characteristics of included patients with a diagnosis of osteosarcopenia (*n* = 42).

Parameter	Value
Age (years, mean ± sd)	69.3 ± 11.3
Gender (Female *n*, %)	37 (88.1%)
Smoker (yes) (*n*, %)	11 (26.2%)
BMI (kg/m2) (mean ± sd)	24.6 ± 3.5
MNA-SF (mean ± sd)	11.4 ± 2.3
ALM (absolute value, kg) (mean ± sd)	13.2 ± 4.2
HGS (kg) (mean ± sd)	14.2 ± 5.5
TBS (mean ± sd)	1,197.2 ± 153.8
BPI-SS >0 (*n*, %)	33 (78.6%)
SPPB (Total value) (mean ± sd)	5.4 ± 2.6
SARC-F ≥ 4 (*n*, %)	15 (35.7%)
Patients with previous fracture events (*n*, %)	17 (40.5%)
EQ-5D-3L (mean ± sd)	0.8 ± 0.2
BPI-SS (mean ± sd)	4.05 ± 2.38
Patients with BPI-SS ≤ 4 (*n*, %)	12 (36.4%)
Patients with BPI-SS >4 and <7 (*n*, %)	16 (48.5%)
Patients with BPI-SS ≥ 7 (*n*, %)	5 (15.1%)

BMI, body mass index; MNA-SF, mini nutritional assessment- short form; ALM, appendicular lean mass; HGS, handgrip strength; TBS, trabecular bone score; BPI-SS, brief pain inventory-severity score; EQ-5D-3L, euroQol-5 dimensions −3 levels; SPPB, short physical performance battery; SARC-F, strength assistance with walking raising from a chair climbing stairs and falls; BPI-SS, brief pain inventory- severity score.

The mean ALM was 13.2 ± 4.2 kg, and the mean HGS was 14.2 ± 5.5 kg, consistent with diagnostic criteria for sarcopenia. The mean TBS was 1197.2 ± 153.8, and 17 (40.5%) of the OSP patients reported a personal history of fractures.

Functional assessment showed a mean total SPPB score of 5.4 ± 2.6, indicating severely impaired physical performance. Regarding the SARC-F, only 35.7% of patients scored ≥4, suggesting a relatively lower risk of sarcopenia according to this screening tool.

The mean EuroQoL-5D-3L score was 0.8 ± 0.2 while Pain assessment revealed that 78.6% of patients reported pain (BPI-SS >0), with a mean BPI-SS score of 4.05 ± 2.38. Specifically, 36.4% of patients experienced mild pain (BPI-SS ≤4), 48.5% moderate pain (BPI-SS >4 and <7), and 15.1% severe pain (BPI-SS ≥7).

**Figure 1:**
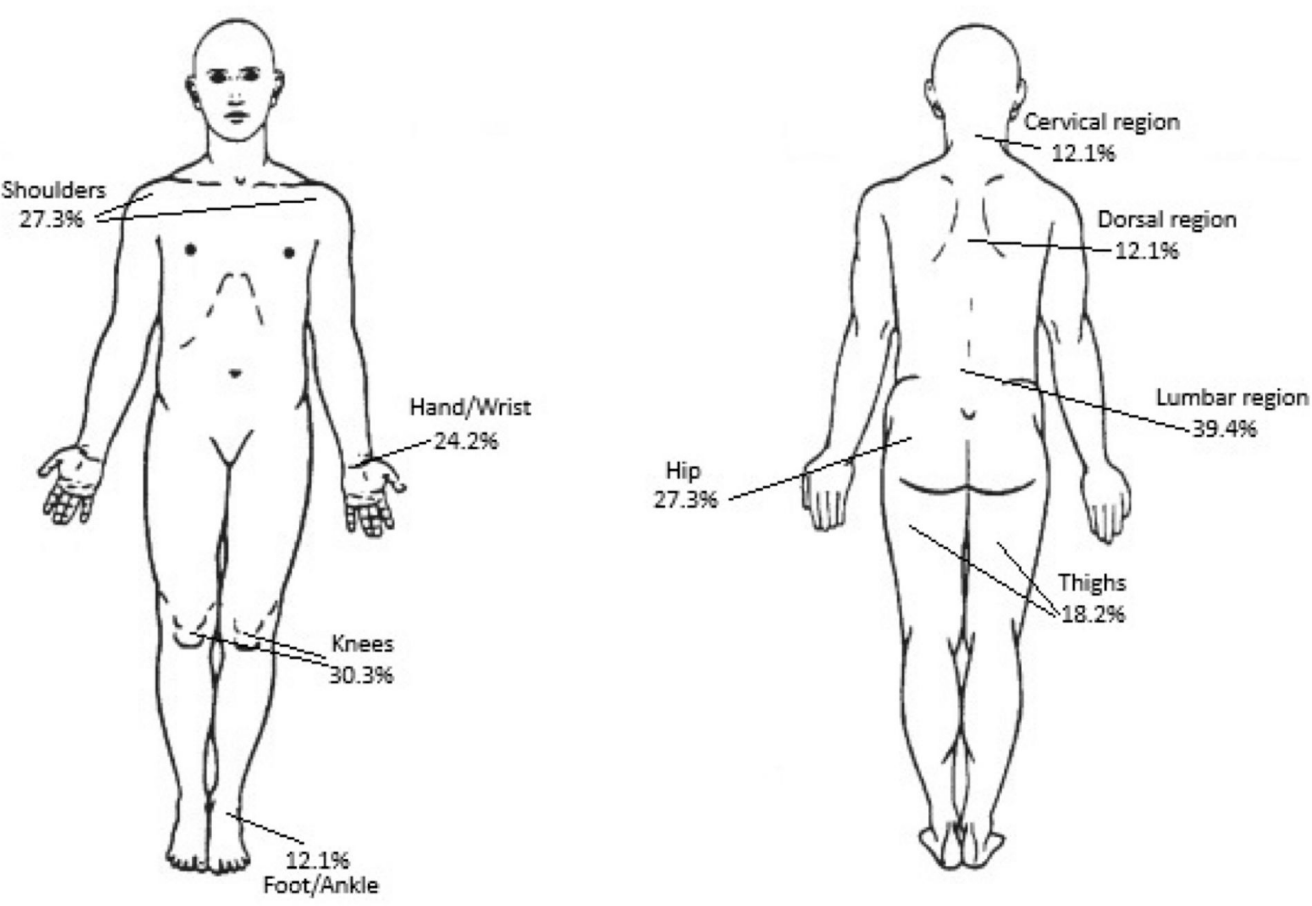
Main sites of pain localization in osteosarcopenic patients: arrows indicate the anatomical region most frequently reported as painful, with percentage representing the proportion of patients.

Pain localization data are shown in Figure 1. Lumbar region was the most frequently reported site (39.4%), followed by the knee (30.3%), and the shoulder and hip (both 27.3%).

Table 2 reports the results of the correlation analysis between pain severity/interference and the other outcome measures age-adjusted. BPI-SS was positively correlated with BPI-IS (*p* = 0.0002), SARC-F (*p* = 0.022) and negatively correlated with ALM (*p* = 0.047), total SPPB score (*p* = 0.0008), MNA-SF (*p* = 0.034), and two SPPB subcomponents [balance (*p* = 0.003) and gait speed (*p* = 0.007)]. These findings suggest that increased pain severity was correlated with increased risk of sarcopenia and pain interference on activity. On the other hand, increased pain severity was correlated with reduced lean mass, poorer nutritional status, impaired balance, slower gait speed, and more difficulty in performing postural changes. Similarly, increased pain interference was positively correlated with SARC-F (*p* = 0.002) and BPI-SS (*p* = 0.0002), and negatively correlated with balance (*p* = 0.0004), gait speed (*p* = 0.001), and total SPPB score (*p* = 0.0003), this last indicating that greater pain interference was correlated with worsening of motor performance.

**Table 2:** Age-adjusted spearman correlation coefficients between pain severity and interference (respectively, BPI-SS and BPI-IS) with appendicular lean mass (ALM), risk of sarcopenia (SARC-F), gait speed, balance, physical performance (SPPB) and nutritional assessment (MNA-SF) in OSP patients.

Parameter	Spearman test	BPI-SS (*n* = 33)	BPI-IS (*n* = 33)	ALM (*n* = 33)	SARC-F (*n* = 33)	Balance (0–4) (*n* = 33)	Gait speed (0–4) (*n* = 33)	SPPB (Total) (*n* = 33)	MNA-SF (*n* = 33)
BPI (S.S.)	Spearman Coefficient		**0** **.** **604**	**−0**.**353**	**0**.**403**	**−0**.**505**	**−0**.**463**	**−0**.**563**	**−0**.**375**
	Significance (2-tailed)	1.00	**0**.**0002**	**0**.**047**	**0**.**022**	**0**.**003**	**0**.**007**	**0**.**0008**	**0**.**034**
BPI (I.S.)	Spearman Coefficient	**0**.**604**		−0.305	**0**.**525**	**−0**.**584**	**−0**.**537**	**−0**.**597**	−0.166
	Significance (2-tailed)	**0**.**0002**	1.00	0.088	**0**.**002**	**0**.**0004**	**0**.**001**	**0**.**0003**	0.363

Bold text represents the statistically significant results.

In patients without fractures, pain severity (BPI-SS) showed a significant moderate positive correlation with SARC-F (*r* = 0.650, *p* = 0.005), and a moderate inverse correlation with ALM (*r* = −0.517, *p* = 0.005), balance (*r* = −0.517, *p* = 0.042), SPPB total score (*r* = −0.521, *p* = 0.04), and MNA-SF (*r* = −0.517, *p* = 0.049) (Table 3). Analysis of BPI-IS revealed a moderate inverse correlation with balance (*r* = −0.650, *p* = 0.007), gait speed (*r* = −0.524, *p* = 0.030), and SPPB total score (*r* = −0.586, *p* = 0.018) (Table 3).

**Table 3:** Age-adjusted spearman correlation coefficients between pain severity and interference (respectively, BPI-SS and BPI-IS) with appendicular lean mass (ALM), risk of sarcopenia (SARC-F), gait speed, balance, physical performance (SPPB) and nutritional assessment (MNA-SF) in older adults with and without fragility fractures.

Groups	Parameters	Spearman test	BPI-SS	BPI-IS	ALM	SARC-F	Balance	Gait-Speed (0–4)	SPPB (Total)	MNA-SF
GROUP WITHOUT FRACTURES (*n* = 25)	BPI (S.S.)	Spearman Coefficient		**0** **.** **599**	**−0**.**517**	**0**.**650**	**−0**.**517**	−0.481	**−0**.**521**	**−0**.**517**
		Significance (2-tailed)	1.00	**0**.**012**	**0**.**049**	**0**.**005**	**0**.**042**	0.061	**0**.**04**	**0**.**049**
	BPI (I.S.)	Spearman Coefficient			−0.392	**0**.**483**	**−0**.**650**	**−0**.**524**	**−0**.**586**	−0.183
		Significance (2-tailed)		1.00	0.14	**0**.**050**	**0**.**007**	**0**.**03**	**0**.**018**	0.476
GROUP WITH FRACTURES (*n* = 17)	BPI (S.S.)	Spearman Coefficient		**0**.**512**	−0.172	0.109	−0.199	−0.358	−0.417	−0.172
		Significance (2-tailed)	1.00	**0**.**024**	0.49	0.65	0.41	0.131	0.07	0.49
	BPI (I.S.)	Spearman Coefficient			−0.293	0.348	−0.20	**−0**.**573**	−0.294	−0.35
		Significance (2-tailed)		1.00	0.23	0.131	0.408	**0**.**010**	0.220	0.131

Bold text represents the statistically significant results.

Conversely, in patients with fractures, pain severity (BPI-SS) demonstrated a moderate positive correlation only with BPI-IS (*r* = 0.512, *p* = 0.024), while BPI-IS showed a moderate inverse correlation with gait speed (*r* = −0.573, *p* = 0.010) (Table 3). Other correlations, including those with ALM and functional performance measures, were weak or non-significant (Table 3).

## 4 Discussion

The present study aimed to characterize the frequency and intensity of pain in a cohort of OSP patients highlighting the central role of musculoskeletal pain as a critical issue in this population. To our knowledge, this is the first preliminary study addressing pain and its correlation with clinical parameters in OSP patients.

The main findings reported a pain frequency in three out of four OSP patients, predominantly localized in the lumbar region, with moderate to severe intensity in over 60% of cases. These data are supported by a recent systematic review reporting that sarcopenic patients are more prone to experiencing higher levels of pain (28).

Our findings provide important insights into the relationship between pain and functional outcomes in osteosarcopenic patients. Pain severity demonstrated a moderate positive correlation with SARC-F, suggesting that higher pain levels are correlated with greater self-reported sarcopenia risk. Conversely, pain severity showed moderate inverse correlations with balance, gait speed, and overall physical performance. These findings indicate that pain severity may contribute to impaired mobility and functional decline, consistent with previous evidence linking chronic pain to reduced physical activity and balance deficits. Similarly, pain interference exhibited even stronger inverse correlations with balance, gait speed, and overall physical performance, highlighting the detrimental impact of pain on functional independence. The positive correlation between pain interference and SARC-F further supports the interplay between pain interference and sarcopenia-related limitations (*p* = 0.002). Interestingly, correlations with muscle mass and nutritional status were weaker and less consistent, suggesting that pain may primarily affect functional rather than structural components of musculoskeletal health.

The observed correlations highlight distinct patterns between patients with and without fractures, suggesting that pain severity interacts differently with sarcopenia-related measures and functional performance depending on fracture status. In patients without fractures, higher pain severity (BPI-SS) was moderately correlated with poorer muscle function and nutritional status, as indicated by positive correlation with SARC-F and inverse correlations with ALM, balance, SPPB total score, and MNA-SF. These findings imply that pain may exacerbate functional decline and nutritional vulnerability in this subgroup, potentially contributing to frailty progression.

Interestingly, BPI-IS also showed moderate inverse correlation with balance, gait speed, and overall physical performance, reinforcing the notion that pain interference negatively impacts mobility and functional independence. This aligns with previous evidence linking chronic pain to reduced physical activity and impaired balance control.

Conversely, in patients with fractures, the relationship between pain and functional measures was markedly weaker. Pain severity correlated only with pain interference (BPI-IS), and BPI-IS showed an inverse correlation with gait speed, while other correlations were non-significant. This suggests that in the context of fractures, pain may be more localized and less predictive of global functional decline, possibly because fracture-related immobility overshadows the influence of pain on sarcopenia and nutritional status. These findings underscore the importance of addressing pain management as part of comprehensive strategies to preserve muscle function and mobility, particularly in patients without fractures, where pain appears to play a more systemic role in functional deterioration.

The lack of correlation between TBS, BMD, and pain seems to suggest that bone quality and density may not be primary determinants of pain in this population. Instead, the significant correlation with sarcopenia parameters highlights the potential role of muscle health in pain perception and functional impairment. This finding aligns with emerging evidence indicating that reduced muscle mass and strength can contribute to altered biomechanics, increased joint stress, and chronic low-grade inflammation, all of which may amplify pain. Particularly, musculoskeletal pain reported by OSP patients may be attributed to the presence of low-grade chronic inflammation. Damage to bone and muscle tissues can initiate an inflammatory cascade driven by the activation of immune cells. These cells release mediators such as IL-1β, IL-6, and TNF-α, which activate ion channels and voltage-gated sodium channels. Notable examples of these channels include the transient receptor potential cation channel subfamily V member 1 (TRPV1), the transient receptor ankyrin 1 (TRPA1), and voltage-gated sodium channels (NaV) found on nociceptive neurons (29, 30). This may promote depolarization and the transmission of pain signals, with further neuropeptide release amplifying inflammation. Peripheral sensitization lowers pain thresholds and contributes to chronic pain (9, 31). This maintained phenomenon, through a bottom-up mechanism, might trigger central sensitization, a key event in the pathophysiology of chronic pain, defined as a process in which the central nervous system becomes hyperactive, amplifying and perpetuating the perception of pain (32, 33). Considering these aspects, implementing a targeted management strategy for the musculoskeletal system, encompassing both pharmacological and non-pharmacological interventions, such as therapeutic exercise, appears to be a promising approach for this population.

While the type and intensity of pain in osteoporosis and sarcopenia have been previously discussed, little is known about its prevalence and impact in patients with OSP. Previous studies have analyzed various clinical and functional outcomes in OSP (7, 10, 34–37), but there is limited evidence characterizing OSP-correlated pain. Our study revealed an inverse correlation between the severity of OSP-related pain, nutritional status, and physical performance, suggesting that pain is not merely a symptom but a critical factor that exacerbates disability and reduces QoL. In line with our findings, Buyukavci et al. reported the presence of pain in a cohort of osteopenic/osteoporotic patients with sarcopenia, highlighting an increased risk of balance loss, fractures, and a decline in QoL (38). However, they did not perform a specific pain characterization, reporting only the pain item from the SF-36. Our study highlights the significant burden of chronic pain in patients with OSP, emphasizing its correlation with muscle mass, nutritional status, and physical performance. The correlation between pain severity and impaired motor skills, including balance and gait speed, underscores the need for early and comprehensive management strategies, including pain treatment, nutritional support, and therapeutic exercise (39).

Being an exploratory study aimed at identifying the limits of correlation research between pain and functional parameters in individuals with OSP, this work lays the groundwork for further investigations on a larger sample and, potentially, with a control group. Another limitation of our study is the unbalanced sex distribution, with a higher number of female than male participants with OSP.

Future research should investigate targeted interventions also to reduce chronic low-grade inflammation, with the ultimate goal of improving functional, preventing disability, and enhancing overall well-being in individuals with OSP.

## 5 Conclusions

This study highlights the high frequency and clinical relevance of pain in individuals with osteosarcopenia (OSP), where osteoporosis and sarcopenia coexist. Pain is moderately correlated with poorer physical performance and indicators of sarcopenia risk, rather than bone parameters. Findings suggest that sarcopenia plays a key role in pain perception and its functional impact. Early interventions targeting muscle health, through nutrition, exercise, and pain management, are essential to reduce pain, falls, and fractures. Comprehensive strategies can improve quality of life and prevent complications in this vulnerable population.

## Data Availability

The raw data supporting the conclusions of this article will be made available by the authors, without undue reservation.
